# The Essential Function of the Health Visitor

**Published:** 1920-11-27

**Authors:** 


### The Essential Function of the Health Visitor.

The Hull Medical Officer of Health has prepared reports on the capacity of health visitors to deal with the work, in view of the Health Ministry's dictum that a district large enough for one visitor to work is one with about 400 births a year. The calls upon the health visitor, he says, are becoming so numerous that all the duties other than domiciliary visitation for health purposes should be regarded as of secondary importance. If the health visitor is engaged for part of her time in making inquiries to verify essential particulars given on forms of application for other services, she is bound to lessen the time spent in home visitation, and in the end bound to lose that supervision of the home which is the essential function of the health visitor. Home visiting is a valuable adjunct to the infant-welfare centres, and forms the essential groundwork of all administrative measures intended to alleviate infant mortality.

Looking at the matter from all points of view, 400 births per year in one district is the maximum number for home-visitation purposes for one health visitor. Other authorities have fixed a lower number, as follows:

Liverpool, eighty-six visitors, 200 births per year each health visitor.

Birmingham, eighty-four visitors, 200 births.

Leeds, twenty visitors and seventeen clinic nurses, 200 births.

Manchester, forty-six visitors and eleven clinic nurses, 250 births.

Bradford, sixteen visitors and twenty clinic nurses, 350 births.

Southwark, fourteen visitors, 400 births (about to be reduced to 250).

The Ministry of Health has laid down that about eight visits a year are required on an average during the first year for each infant born. How much visiting does this imply for the health visitor in charge of a district which averages 400 births per annum? Four hundred multiplied by eight equals 3,200. And then there are the visits to be paid to children from the end of infancy to school age. There will probably be something like 1,600 children in this group in each district. Not all of them require visiting, for many of them are members of the same household as an infant. It is difficult, therefore, to estimate the number of visits which must actually be paid to these children, but probably at two visits per annum the number will not be far short of 1,600. To carry out, therefore, the ideas of the Ministry of Health, something like 4,800 visits per annum must be paid, or over eighteen per working day.

